# Nutrient management: as a panacea to improve the caryopsis quality and yield potential of durum wheat (*Triticum turgidum* L.) under the changing climatic conditions

**DOI:** 10.3389/fpls.2023.1232675

**Published:** 2023-08-28

**Authors:** Anteneh Agezew Melash, Amare Assefa Bogale, Bekir Bytyqi, Muhoja Sylivester Nyandi, Éva Babett Ábrahám

**Affiliations:** ^1^ Kálmán Kerpely Doctoral School of Crop Production and Horticultural Science, University of Debrecen, Debrecen, Hungary; ^2^ Department of Horticulture, College of Agriculture and Environmental Science, Debark University, Debark, Ethiopia; ^3^ Institute of Crop Production, Hungarian University of Agriculture and Life Sciences, Gödöllő, Hungary; ^4^ Faculty of Agricultural, Food Sciences and Environmental Management, Institute of Crop Sciences, University of Debrecen, Debrecen, Hungary

**Keywords:** durum wheat, nutrient management, grain quality, yield, enrichment of CO2, drought, water logging, temperature

## Abstract

The increasing human population and the changing climate, which have given rise to frequent drought spells, pose a serious threat to global food security, while identification of high-yielding drought-tolerant genotypes coupled with nutrient management remains a proficient approach to cope with these challenges. An increase in seasonal temperature, recurring drought stress, and elevated atmospheric CO_2_ are alarmingly affecting durum wheat production, productivity, grain quality, and the human systems it supports. An increase in atmospheric carbon dioxide can improve wheat grain yield in a certain amount, but the right amount of nutrients, water, and other required conditions should be met to realize this benefit. Nutrients including nitrogen, silicon, and sulfur supply could alleviate the adverse effects of abiotic stress by enhancing antioxidant defense and improving nitrogen assimilation, although the effects on plant tolerance to drought stress varied with nitrogen ionic forms. The application of sewage sludge to durum wheat also positively impacts its drought stress tolerance by triggering high accumulation of osmoregulators, improving water retention capacity in the soil, and promoting root growth. These beneficial effect of nutrients contribute to durum wheat ability to withstand and recover from abiotic stress conditions, ultimately enhance its productivity and resilience. While these nutrients can provide benefits when applied in appropriate amounts, their excessive use can lead to adverse environmental consequences. Advanced technologies such as precision nutrient management, unmanned aerial vehicle-based spraying, and anaerobic digestion play significant roles in reducing the negative effects associated with nutrients like sewage sludge, zinc, nanoparticles and silicon fertilizers. Hence, nutrient management practices offer significant potential to enhance the caryopsis quality and yield potential of durum wheat. Through implementing tailored nutrient management strategies, farmers, breeders, and agronomists can contribute to sustainable durum wheat production, ensuring food security and maintaining the economic viability of the crop under the changing climatic conditions.

## 1 Introduction

Durum wheat *(Triticum turgidum* L.*)*, a commonly cultivated form of allotetraploid, holds particular significance due to its essential role in the production of semolina, a key ingredient for pasta and macaroni manufacturing (Bin et al., 2017; Andrej et al., 2021). The capacity of durum wheat to produce high-quality foodstuffs is strongly determined by the content and composition of grain storage proteins, which form a viscoelastic network called gluten that is formed when flour is hydrated and mixed into a dough (Koga et al., 2016). This network allows the dough to stretch and retain its shape during pasta and macaroni processing, such as kneading and extrusion. Although durum wheat offers such enormous economic and industrial benefits, the yield, grain protein, and mineral concentration may wane in the future due to changing climatic circumstances (Ben et al., 2021). Globally, durum wheat production accounts for 5% of the total wheat production, cultivated across 16 million hectares of planting area (Beres et al., 2020). However, only 13% of the world’s arable land is suitable for durum wheat cultivation, and as a result of climate change, the suitable area may have decreased by 19% and 48% in the middle and end of the century, respectively (Andrej et al., 2021). These changes could intensify extreme meteorological and hydrological events, including drought, waterlogging, and heat waves, which have also increased persistently in terms of both frequency and intensity (Seneviratne et al., 2012; Yu et al., 2018).

The changing climatic conditions pose a substantial threat to crop production, including durum wheat, as they give rise to significant alterations in phytochemical, physiological, and biochemical processes. These changes can have severe repercussions, impacting both the yield of durum wheat and the surrounding environment. A shift in climatic conditions such as high temperatures and drought stress has turned out to be the most important constraining factor for the crop production sector, where a substantial effect is frequently observed at the later developmental phases (Ben et al., 2021). It has been observed that, up to a certain concentration, atmospheric CO_2_ enrichment could increase yield and grain starch accumulation, but it also negatively affects the nutritional profile of grains, such as the protein and mineral content of most cereals (Asseng et al., 2019; Ben et al., 2021). However, the mechanisms behind these additive and antagonistic effects remain obscure, although common understanding ascribes the dilution effect as the primary cause of the decline in grain nutritional profile (Santos et al., 2023). Identification of drought-tolerant and high-yielding cultivars combined with proper nutrient management could be an effective approach to reduce these challenges (Boudjabi et al., 2015; Ma et al., 2017; Melash et al., 2019). Indeed, this approach may necessitate an understanding of crop response to water stress as well as crop responsiveness to the applied nutrients (Shew et al., 2020).

Identifying the resilience of yield and grain quality under changing climatic conditions is of utmost importance in effectively addressing the challenges posed by CO_2_ enrichment, drought, and high temperature stresses. Hence, understanding the temporal and spatial scales of the consequences of these stressors is important for developing effective adaptation and mitigation strategies. It has been proven that unless global efforts to reduce greenhouse gas emissions are promptly and significantly intensified, the effects of climate change will be more profound on future durum wheat production. This could further constrain the harvestable yield and morphometric traits of wheat, often through a decrease in important yield attribute traits such as, the number of seeds per spike, grain weight, and spike length (Hasan and Tacettin, 2010). However, there has been relatively limited consideration of potential climate impacts on malnutrition through mechanisms such as changing the nutrient content of food products (Santos et al., 2023). These climate-induced shifts impact on suitable growing areas, grain yield, and nutritional composition, necessitating a comprehensive understanding and eventual adoption of efficient and sustainable nutrient management techniques to stabilize production and adapt the entire food supply chain.

Nutrient management emerges as a promising panacea to counterbalance the negative impacts of changing climatic conditions on durum wheat cultivation (Melash and Ábrahám, 2022). Advanced site-specific technologies and techniques allow for the precise customization of nutrient applications, considering factors like timing, rate, and placement, to optimize durum wheat productivity while minimizing environmental impacts (Kizilgeci et al., 2021). The application of precision nutrient management to durum wheat has demonstrated encouraging results, leading to improved grain yield and protein content. Moreover, precision nutrient management not only enhances crop yield and income but also promotes the efficient utilization of nutrients and water while reducing greenhouse gas (GHG) emissions (Sadhukhan et al., 2023). In the broader context of agriculture, implementing nutrient management strategies based on a nutrient expert approach has also shown considerable benefits. This approach has resulted in a significant reduction of 1.44 million tons in nitrogen fertilizer use and a decrease of 5.34 million tons of CO_2_ equivalent emissions annually in addition to increasing yield of crops such as rice and wheat (Sapkota et al., 2021). This reduction reflects a more precise and efficient use of nitrogen inputs, ensuring that crops receive the optimal amount of nutrients, reducing wastage, and mitigating potential environmental impacts. The strategic utilization of nitrogen fertilizer offers a significant potential for enhancing grain yield and bolstering crop stress tolerance. Nitrogen plays a crucial role in maintaining leaf water potential, facilitating photosynthetic activities, and fortifying antioxidative defense mechanisms, thus contributing to crop performance (Abid et al., 2016). In the context of drought stress and associated challenges in wheat crop, the application of silicon, seaweed extracts, sewage sludge, and zinc-containing fertilizers has been found to effectively alleviate the inhibitory effects of abiotic stressors (Boudjabi et al., 2015; Coskun et al., 2016; Ma et al., 2017). Hence, incorporating zinc-containing fertilizers, sewage sludge, and silicon-based nutrients into the cultivation system of durum wheat can provide several benefits including optimizing nutrient availability, promoting plant health, and enhancing the crop’s resilience to changing climatic conditions.

The protagonists of nutrient management technologies have been reported in various studies as a potential agronomic solution to enhance yield and grain quality traits under the current climate change scenarios (Sapkota et al., 2021). However, fertilizer management practices alone cannot single-handedly mitigate climate change effects on durum wheat productivity but rather should be viewed as a vital component of a comprehensive set of climate-smart agricultural strategies. Hence, adopting precision agriculture techniques, integrating organic and inorganic fertilizers, and implementing supportive policies can optimize nutrient use, reduce greenhouse gas emissions, enhance soil carbon sequestration, and promote sustainable agricultural systems. Indeed, scientific verification has shown that the integration of farmyard manure along with additional silicon based fertilizers enhances growth, increases grain yield, improves nutrient uptake, crop quality, and boosts nitrogen-use efficiency of the crop (Naik et al., 2022). Through a comprehensive analysis of existing research this review article aims to achieve two primary objectives. Firstly, it seeks to identify knowledge gaps, challenges, and opportunities associated with fertilizer management in durum wheat production. The article could therefore provide valuable recommendations for future research and policy development in this field, with the ultimate goal of optimizing fertilizer use and improving agricultural practices. Secondly, it aims to examine the effectiveness of nutrient management strategies in enhancing the yield, physiology, and grain nutritional composition of durum wheat under abiotic stress conditions and promoting sustainable agricultural systems. By assessing the outcomes of different nutrient management approaches the article can contribute to the development of sustainable agricultural system that promote resilient crop production. Through these objectives, the article can universally serve as a valuable resource for agronomists, researchers, and farmers alike, facilitating better decision making and fostering advancements in durum wheat cultivation for improved food security and sustainable agricultural practices under the changing climatic scenarios.

## 2 Methodology

A comprehensive search of the literature has been conducted to identify relevant studies and publications related to the topic of interest. The search was performed across various electronic databases such as PubMed, Scopus, Web of Science, and Google Scholar. Keywords and terms used in the search included the terms climate change, drought, waterlogging, durum wheat varieties in combination with the terms qualitative and qualitative traits. The initial search yielded a total of 236 articles, which were then screened based on their titles and abstracts for relevance to the topic under investigation. After the initial screening, a more detailed evaluation of the selected articles was conducted and included studies that met the following inclusion criteria: online accessibility, written language, and most importantly, primary data. Studies that did not meet the inclusion criteria or were duplicates were excluded. The full texts of the selected articles were obtained and reviewed for their relevance to the research question and the quality of the methodology employed.

The selected studies encompassed a wide range of experimental designs, including randomized controlled trials, long-term observational studies, and field and greenhouse experiments as well. In order to provide a comprehensive analysis, studies investigating the effects of various fertilizer types, including synthetic fertilizers, organic amendments, and bio-fertilizers, and their role under changing climatic conditions were included in this review. The overall findings of the included studies are summarized and presented in a narrative format, highlighting the key trends, patterns, and knowledge gaps in the literature.

## 3 Building a resilient future: evaluating the resilience of yield and grain quality in a changing climate

### 3.1 Unveiling the impact of drought-induced stress on grain yield and quality

**Figure 1:**
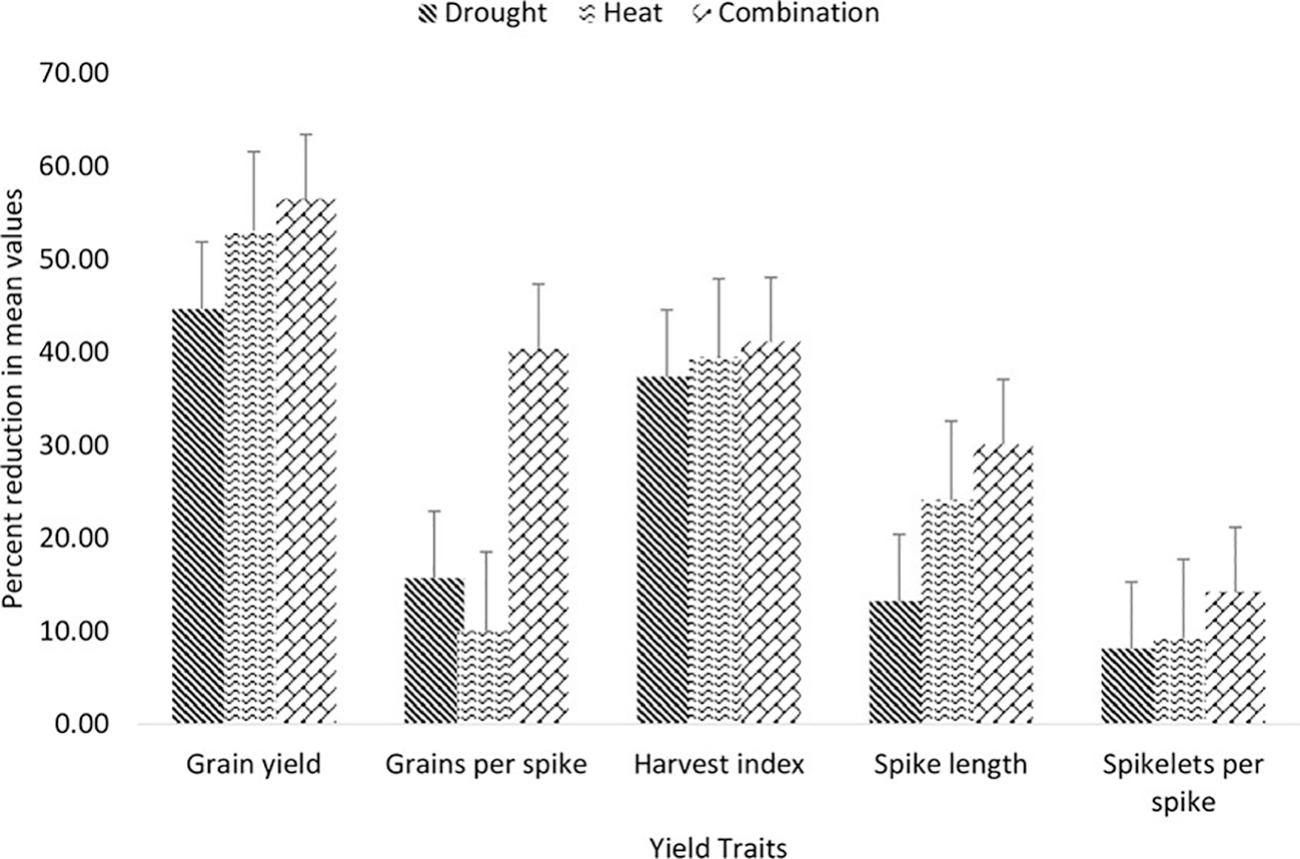
illustrates a percentage reduction in grain yield and associated traits of wheat genotypes evaluated under drought, heat, and combinations of both stress treatments (Qaseem et al., 2019).

The change in rainfall pattern could increase short-run crop failures and cause long-run reduction in production. This is primarily due to the adverse effect it can have on morphometric characteristics and associated yield-attributed traits of crops. The harmful effect of drought stress on grain yield can be significantly amplified when it occurs with the presence of various other climatic parameters, as illustrated in **Figure 1** (Pradhan et al., 2012; Qaseem et al., 2019). Its cohabitation and concurrent effect can have a synergistic, antagonistic, or hypo-additive effect on yield and other associated yield attribute traits (Prasad et al., 2011). Heat and drought-induced stress significantly affect the rate of wheat growth and development, and under such conditions, wheat species could complete their developmental cycle much faster than under normal conditions (Ghazi, 2012). However, the crop might have a short duration with fewer days to accumulate more assimilates during their entire developmental cycle (Erda et al., 2005; Wahid et al., 2007). This effect initiates various physiological processes, such as a decreased rate of photosynthesis coupled with abnormal respiration, stomata closure, and high leaf temperature, leading to a diminished potential for biomass production (Mittler, 2006; Qaseem et al., 2019). Hence understanding the effect of heat and drought stress on durum wheat growth and development is crucial for developing strategies to mitigate their negative impacts and ensure sustainable production in changing environments.

The austerity and negative impact instigated by drought stress are usually unforeseeable, as they are also controlled by multiple factors, including patterns in rainfall, the water-retaining ability of the soil, and a water deficit due to a high crop transpiration rate (Yan et al., 2016). The combination of these factors contributes to the complex nature of drought and its impact on the entire agricultural system. These situations further aggravate the drought stress and influence the overall productivity of crops by affecting the relationships with water-soluble nutrients and eventually causing a substantial decline in grain yield due to the impairment of the photosynthetic process (Rakshit et al., 2020; Gebrewahid et al., 2021). These results universally indicate that drought stress is not solely determined by absence or deficiency of rainfall; it also depends on how the available water is retained in the soil and the rate at which the crop consume water through transpiration. Although grain yield is a multifarious amalgamation of diverse yield-attributed traits, drought-mediated yield reduction could account for up to 50% of the total grain production (Ben et al., 2021). Pollen abortion, a decrease in the amount of conserved food, and the formation of sterile tillers are the main factors contributing to a decrease in grain formation during dry climatic conditions (Duggan et al., 2005; Sinclair and Jamieson, 2006; Ji et al., 2010). The higher deposition of abscisic acid in the spike, triggered by drought stress, has also been found to potentially reduce the pollination capacity of the ovary, leading to significant seed abortion and a decline in seed set (Weldearegay et al., 2012). These results indicate the importance of developing improved durum wheat varieties that possess enhanced resistance to drought stress; thus, the utilization of novel molecular markers and their successful integration into breeding programs is a valuable approach to achieving maximum production.

Water stress during the critical growth stages of wheat, such as flowering and grain filling, can be particularly detrimental. These stages are crucial for the development and filling of grains, and any water shortage during this time can result in reduced morphometric traits, such as plant height, the number of tillers, biomass yield, and grain weight, which could decrease along with the grain filling rate (Nouri et al., 2011). A substantial decline in leaf area and photosynthesis activity was also detected under drought stress, which eventually intensified to reduce the number of leaves per plant, leaf size, and longevity (Shao et al., 2008). This could be due to the limitation of leaf extension under water stress conditions to balance the absorbed water by the root and the water status of plant tissue (Nezhadahmadi et al., 2013). While the effect of drought stress is a complex issue, the specific outcomes can vary depending on various factors, such as the duration and intensity of drought events, plant health conditions, nutritional status, varietal differences, and growth stages at which crops are exposed to drought and the environmental conditions in which crops are grown (Sun et al., 2017; Yu et al., 2018).

#### 3.1.1 Drought stress and grain protein content: understanding the complex relationship

The grain protein content plays a multifaceted role, particularly in relation to both drought conditions and in the context of food products. Higher grain protein concentration is not only improving the quality of end-use products but also enhance survival of the cells against stress conditions due to its role in stabilizing the membranes (Jamshidi et al., 2020). Higher protein content in grains can help crops maintain metabolic functions, sustain growth, and enhance drought tolerance, as proteins are involved in various physiological processes, including enzymatic reactions, cellular signaling, and stress responses (Merewitz et al., 2011). However, establishing a clear and consistent relationship between drought stress and grain quality attributes in durum wheat has indeed posed challenges, despite substantial research efforts (Flagella et al., 2010; Gebrewahid et al., 2021). The absence of a “one-size-fits-all” model for the impact of drought stress on durum wheat, could be a reflection of the complex nature of drought stress and the varied responses observed across different crop ecotypes and genotype-by-environment (G × E) interactions. The presence of additional factors and methodological differences among previous studies can contribute to the challenge of establishing a simple one-dimensional model for drought stress alone and make it difficult to consolidate research findings into a single model.

The relationship between drought and grain protein content could vary depending on the durum wheat varieties, the pedoclimatic conditions of the growing environment, and their interaction (Melash and Ábrahám, 2022). The interactions and synergistic effects between these factors further complicate the prediction and management of drought stress. In some cases, drought stress significantly decreases the grain protein content, to an extent that varies with the degree and timing of the drought events as well (Flagella et al., 2010; Gebrewahid et al., 2021). Gene expression of storage protein fractions such as gliadin, glutenin, -gliadin, and -gliadin has also been downregulated when the cropping season experiences a dry spell (Yang et al., 2011; Begcy and Walia, 2015). The decreased grain protein concentration in water-limited environments can be primarily attributed to the limited availability and assimilation of nitrogen, which is an essential component of storage grain proteins (Zia et al., 2021). This negative consequence of the drought effect, along with poor caryopsis quality, could further constrain the strength of the dough and its stability, such as loaf volume and valorimetric values (Tsenov et al., 2015).

While a number of studies suggest a negative effect of drought stress on durum wheat grain protein content, there are also controversial findings and alternative perspectives. It has been observed that grain protein content can be significantly ameliorated when wheat crops are subjected to drought stress (Flagella et al., 2010; Ahsan et al., 2022). The improved grain protein content and associated quality traits under such a scenario could be due to the reduction in grain starch accumulation (Barnabas et al., 2008), and the limited starch accumulation allows for a higher concentration of nitrogen per unit of starch in the grains (Stone and Nicolas, 1998). The decreased starch accumulation could be due to a decrease in amylose composition, which causes the loose packaging in the starch granules (Prathap et al., 2019). The loss of packaging in the starch granules can further influence grain functional properties, such as the ability to form gels and thickening properties, in food and industrial applications. Additionally, the shortening of grain filling stages under drought conditions could also result in reduced starch accumulation in the developing grains due to early senescence of the crop (Prathap et al., 2019). Early senescence in crops results in a reduction in their photosynthetic capacity of crops, which further exacerbating the limited supply of assimilates required for grain filling and starch synthesis (Sehgal et al., 2018). It is, therefore, very important to consider all involved production factors when studying and managing the impact of drought stress on grain protein content to develop targeted mitigation strategies and breeding programs aimed at improving protein content under water-limited environmental conditions.

#### 3.1.2 Drought stress affects nitrogen assimilation to result in poor phytochemical composition

When evaluating the effect of drought stress on durum wheat productivity, it is important to consider various factors that directly influences overall crop performance. Impairment in symbiotic nitrogen fixation under drought conditions has been observed due to improvement in oxygen diffusion resistance in root bacteroides; resulting in reduced nitrogenase activity that may potentially decrease the availability of nitrogen for the biosynthesis of proteins (Sehgal et al., 2018). There is convincing evidence that the change in composition of protein subunits owing to drought and seasonal heat stress is principally due to alterations in the amount of accumulated nitrogen at the grain filling stage (Triboï et al., 2003; Urban et al., 2018). Investigating the impact of drought stress and associated extreme events under a range of nitrogen doses on physiological traits and other attributes could provide important insights in the development of drought tolerant wheat varieties (Teixeira et al., 2014). This indicates that, although grain protein quality largely depends on the varietal performance, it may be affected by environmental-induced factors (Gebrewahid et al., 2021).

The reduction in mineral accumulation such as iron, zinc, nitrogen, phosphorus, and total protein content in developing grains due to drought stress has also been observed in a wide range of crops (Sehgal et al., 2018). The low phytochemical composition observed in crops under drought stress is often characterized by lower levels of secondary metabolites such as phenolics, flavonoids, alkaloids, and terpenoids. These compounds play crucial roles in crops defense against various environmental stressors, as well as in providing health benefits to humans when consumed as part of a plant-based diet (Bindu et al., 2021). This can have implications for plant growth, development, drought tolerance, and the nutritional and therapeutic properties of plants. Hence, understanding these effects is important for crop management practices and the development of strategies to mitigate the impacts of drought on the physiology and phytochemical composition of durum wheat.

While there is general knowledge about the importance of nitrogen in growth development and phytochemical composition of crops, the intricate interactions between variable nitrogen rate, nitrogen source, drought stress, and specific phenological phases of durum wheat are still an active area of research. An investigation that examines the impact of drought stress under variable nitrogen doses on resource use efficiency, physiological traits, and other associated traits can provide valuable insights for developing drought-tolerant crop varieties (Teixeira et al., 2014). Hence, conducting a comprehensive evaluation of the combined effect of nitrogen deficiency and drought stress can provide a valuable insights into the physiological, biochemical, and phytochemical responses of durum wheat varieties to drought stress. This evaluation can also help elucidate how these responses interact with nitrogen availability, thus agronomists, and durum wheat breeders can develop crop varieties that are more resilient and adaptable to the changing climatic conditions.

### 3.2 Unravelling the heat puzzle: decoding the impact of high temperature stress on crop yields

Higher temperature stress eventually reduces harvestable yield while encouraging weed and pest proliferation. In most cases, crops could respond to high-temperature stress in two different phases. The first stage is based on the intrinsic tolerance to high temperature-induced damage, known as basal thermos-tolerance, while the second stage involves resource mobilization and gene expression changes to deal with heat stress related injury, known as acquired thermos-tolerance (Bento et al., 2017). High temperatures have been observed to cause disruptions in the structure and function of chloroplasts, a reduction in chlorophyll content, and the inactivation of chloroplast enzymes, resulting in decreased photosynthesis activity in wheat (Yang et al., 2002). Increased temperature stress during the reproductive stage may also have an impact on spike fertility and, as a result, grain yield (De Storme and Geelen, 2014). It has been worth mentioning that pollen formation in wheat is a heat-sensitive process, and high temperature-induced pollen sterility often occurs due to irregularities during microsporogenesis (Jäger et al., 2008; Narayanan, 2018).

**Figure 2:**
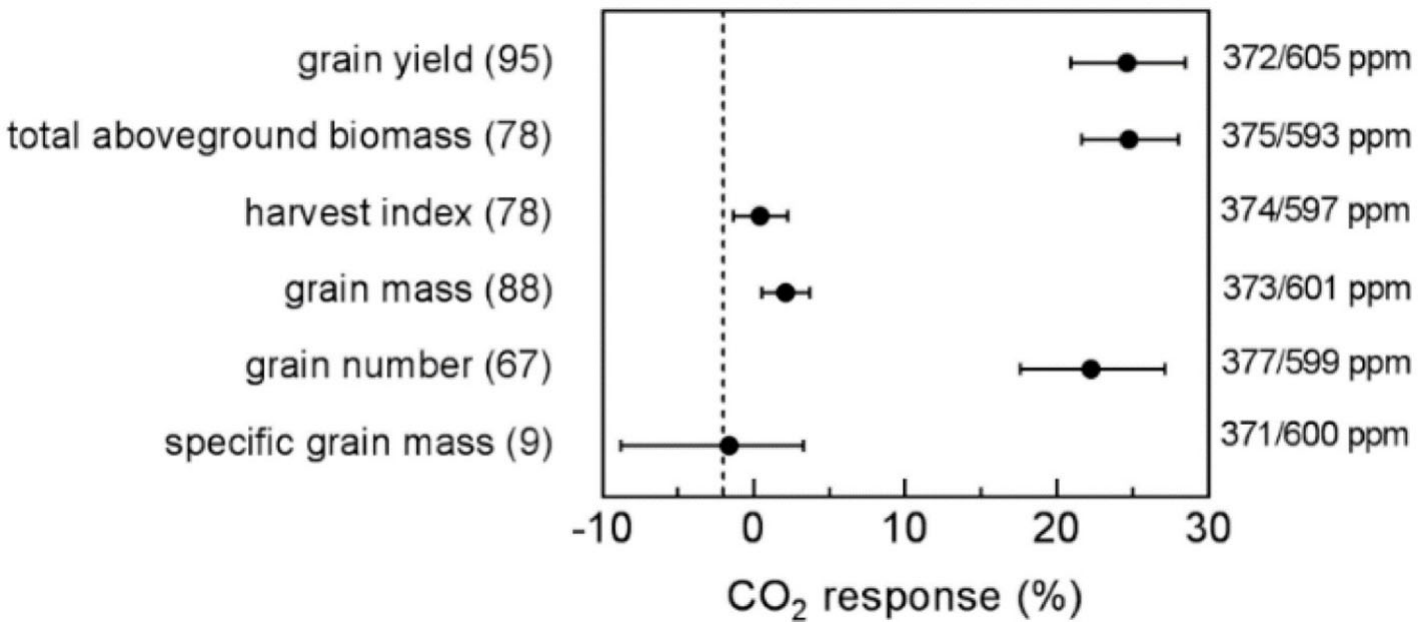
The eCO2 effect on wheat yield components (grain yield, harvest index, grain mass, grain number, total aboveground biomass, and specific grain mass) using aCO2 as the reference. The number of observation pairs is given within the brackets. The average concentration level for ambient and elevated CO2 treatments is given on the right y-axis (aCO2/eCO2).

While elevated carbon dioxide levels can influence certain aspect of crop performance, it’s overall effect on physiology, grain quality, and yield, the overall effect is universally considered to be smaller compared to the challenges posed by high-temperature stress (Ben et al., 2021) (**Figure 2**). This effect triggers a decline in global wheat production of about 4.1 to 6.4% for each degree escalation in temperature (Liu et al., 2016; Ashwani et al., 2020). Wheat crops grown under warmer climatic conditions are more susceptible to significant grain yield losses than wheat cultivated in cooler climatic conditions (Sommer et al., 2013). However, it is worth noting that there is general consensus that in high latitude regions, spring wheat production would benefit from a warmer climate through an extension of the growing season (Sommer et al., 2013). such a huge grain yield loss under warmer or dry climatic conditions could be due to negative water and energy balances resulting from limited water availability and imbalanced energy inputs, which can offset the positive effect of elevated CO_2_ on stomatal conductance, which leads to net losses in soil water content, affecting wheat physiology and eventually grain yield (Helman and Bonfil, 2022). The potential losses in grain yield associated with warmer climatic conditions in wheat crops necessitate careful consideration of climate change impacts and the implementation of appropriate management practices and breeding strategies.

In certain situation, the combination of reduced cooling caused by lower transpiration rates and an increased leaf area index (LAI) can lead to unexpectedly higher water losses in plants (Helman and Bonfil, 2022). In a wet growing environment, varieties with a higher LAI and radiation use efficiency could benefit from the increased availability of water and solar radiation. A higher LAI allows for greater light interception and photosynthetic activity, leading to increased biomass production and subsequently higher number of grain and yield. On the other hand, under hot and dry climatic conditions, short maturing varieties with high grain dry-matter potential and stay-green capacity tend to perform better (Padovan et al., 2020). The shorter maturation periods could allow the crop to complete their life cycle prior to the onset of server stress, while grain dry-matter potential ensures efficient utilization of available resources for grain production. Hence, understanding the genetic basis for variation in phenology and other adaptive morphometric traits could enable wheat breeders and agronomists to predict grain yield risk factors, such as drought and heat, and thereby improve agronomic crop management practices.

### 3.3 Enrichment of atmospheric carbon dioxide effect on qualitative and quantitative agronomic traits

The composition of durum wheat grain encompasses various nutrients, such as proteins, carbohydrates, minerals, and phytochemicals, which collectively contribute to its nutritional and market value of the harvested product (Melash and Ábrahám, 2022). This diverse array of components collectively contributes to the nutritional quality and market value of harvested durum wheat. The concentration can be influenced by a number of factors such as nitrogen availability and environmental conditions. Lower carbon dioxide (CO_2_) concentrations, for instance, has been shown to have an impact on the physiology and metabolome of mature grains. These changes in grain nutrient composition can subsequently affect the nutritional status of the grain (Wang J. et al., 2021). Higher CO_2_ levels can increase morphometric traits and grain yield in crops, primarily due to their positive effect on stimulating photosynthetic activities (Erda et al., 2005). It has been proven that an increment in CO_2_ up to 550 ppm (parts per million) can consistently increase both biomass and grain yields by about 5–15% (Ainsworth and Long, 2005). However, a nonlinear response to elevated carbon dioxide levels has been observed in some studies, where the stimulation of grain and biomass yield starts to diminish at around 600 ppm of CO_2_ (Fitzgerald et al., 2016). This nonlinear response suggests that there might be a saturation point beyond which further increases in CO_2_ do not provide additional yield benefits in crops; although the specific threshold at which this occurs can vary depending on crop ecotype, temperature and seasonal water availability

Increased atmospheric CO_2_ levels and their interactions with other production limiting factors, such as water availability and nitrogen supply, can strongly modulate crop growth responses and result in a wide range of growth responses, typically spanning between 0% and 50% (Erda et al., 2005). The coexistence of elevated carbon dioxide and high temperature stress can have negative effects on wheat varieties, leading to reduced biomass and grain yield, perhaps due to a reduction in the number of spikes per plant (Galani et al., 2022). This implies that while elevated CO_2_ levels can initially stimulate photosynthesis, enhance grain yield and biomass production, the combined effects of heat and drought stress can override these benefits and lead to reduced grain yield (Ben et al., 2021). It means that the combined effect of multiple abiotic stress factors can be more detrimental to durum grain protein content and yield than individual stressors alone.

**Figure 3:**
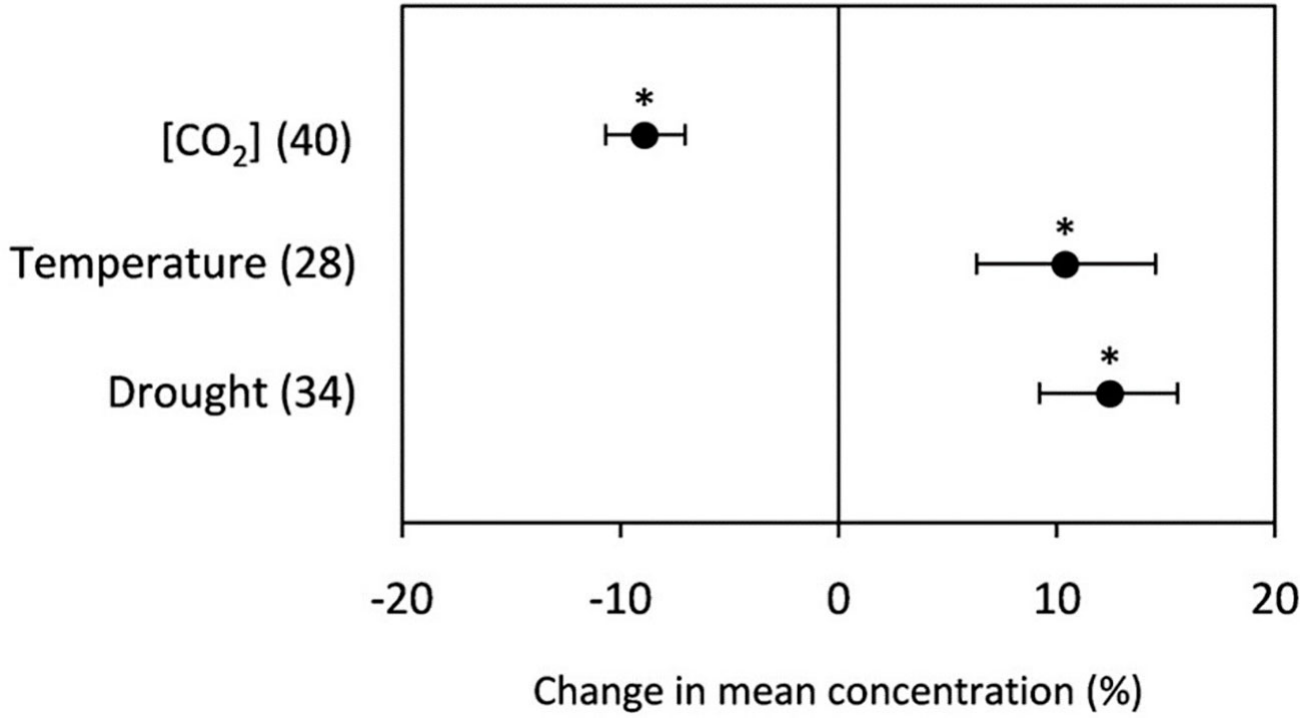
The change in grain protein content of wheat grown under high temperatures, elevated CO2, and drought stress conditions The numerical data in the parentheses indicates, the number of observations, and the error bars indicate the 95% CI, while the single asterisk (*) indicates a statistically significant difference between the observations at p < 0.05.

The detrimental impact of elevated CO_2_ on crop grain yield has been extensively studied (**Figure 3**), shedding light on the intricate mechanisms and physiological responses involved. However, little is known about how it may affect the nutritional composition of wheat grains, despite the fact that it is a vital aspect of food security (Haddad et al., 2016; Asseng et al., 2019). The changes in chemical composition observed in crops, such as a decrease in leaf nitrogen concentration and an increase in the carbon-to-nitrogen (C/N) ratio, could be strongly associated with increasing CO_2_ concentration (Wieser et al., 2008; De Santis et al., 2021). This effect is thought to occur because, as CO_2_ levels increase, crops can more easily convert the excess carbon into carbohydrates, which can lead to a dilution of protein levels in the grain. When grain protein content is reduced under elevated CO_2_ levels while total yield production remains relatively constant, there is a dilution effect, resulting in lower grain protein content on a per-unit basis (Thompson et al., 2019). There has been an estimated decrease in grain protein content of about 1.08% for each 1 t ha^-1^ yield increment under elevated CO_2_ conditions (Galani et al., 2022). It means that as crop yields increase, there is a corresponding decrease in grain protein content, particularly under elevated CO_2_ conditions. This could have significant implications for food security and the market value of the product, as protein is an important nutrient for human health and industrial purposes; thus, it is an important factor to consider in breeding and food production programs (Melash and Ábrahám, 2022).

### 3.4 Dynamic in quantitative and qualitative agronomic traits under waterlogging stress

Water logging, which occurs when soil becomes water-saturated and oxygen is limited, can have a number of negative effects on crop growth, development, and grain protein content (Ren et al., 2014; Chao et al., 2022). This is because waterlogging can reduce the availability and uptake of nitrogen by plants, which is an essential nutrient for the production of storage proteins (Otie et al., 2019). When plants are waterlogged, the roots may not be able to take up nitrogen from the soil (Kaur et al., 2020a), or the nitrogen in the soil may be transformed into a form that is not readily available to plants. As a result, the plants may not be able to produce as much protein, or the protein content they do produce may be of lower quality. This can have a negative impact on the nutritional quality and commercial value of the grain. However, enhanced grain protein content has been observed, while starch concentration was decreased under waterlogging conditions occurred at maturity (Wang J. et al., 2021). Higher grain protein content under such conditions could be due to inhibition of carbohydrate transformation into starch in the developing wheat grain (Zhou et al., 2018). This means that, although an increase in grain protein content is a possible response, it is not always consistent under waterlogged conditions.

When durum wheat experiences water logging, the crop undergoes a series of physiological and biochemical changes to cope with the existing stress conditions. These changes often include alterations in physio-morphological traits, such as biomass production, grain weight, and photosynthetic activity, due to restrictions in the availability of oxygen to the roots (Arguello et al., 2016). A decrease in photosynthesis could limit the ability of crops to produce energy and assimilate carbon dioxide, resulting in a slower growth rate and a lower harvestable yield. Waterlogging situations can also induce the formation of adventitious roots in durum wheat (Tian et al., 2021). These roots arise above the waterlogged zone and serve as a strategy to overcome oxygen deprivation in the root zone. A suppressed root respiration, decreased root activity, and energy shortage have also been observed under waterlogged soil as compared to well-drained conditions (Pan et al., 2021). The impaired root system under waterlogged conditions could further limit the ability of crops to absorb essential nutrients, leading to nutrient deficiencies. Inadequate nutrient uptake affects wheat growth and reduces shoot growth, ultimately resulting in grain yield loss (Herzog et al., 2016). Hence, to avert grain yield and quality loss under waterlogging conditions, agronomists, plant breeders, and researchers focus on the development of crop varieties with improved tolerance to excess water, such as deep root systems, enhanced photosynthetic efficiency, and better nutrient uptake capacities. Additionally, agronomic practices such as proper drainage, crop rotation, and soil management can minimize the negative impacts of waterlogging on crop productivity.

A switch from aerobic respiration to anaerobic respiration due to waterlogging can also decrease the grain yield by preventing culms from generating spikes, slowing spikelet formation, decreasing the number of spikelets spike^-1^, the formation of florets spikelet^-1^, and the number of kernels spike^-1^ (Pampana et al., 2016). However, the actual yield loss under waterlogging conditions could vary widely depending on the durum wheat varieties, their tolerance to waterlogging, the variation in the growing environment, and the duration and severity of waterlogging (Cotrozzi et al., 2021). In some cases, yield losses could be moderate, ranging from 19% to 30%, while in severe and prolonged waterlogging conditions, yield losses can exceed 55% or even lead to total crop failure (Marti et al., 2015; Pampana et al., 2016). Extended waterlogging conditions and anaerobic respiration could further trigger the accumulation of toxic metabolites such as lactic acid, ethanol, and aldehydes, along with increases in reactive oxygen species, resulting in cell death and crop senescence (Pan et al., 2021). Inhibited gaseous exchange capacity under waterlogging conditions could also cause a rapid buildup or plant hormone degradation and further influence the waterlogging tolerance of crops (Kuroha et al., 2018; Pan et al., 2021).

It has been globally estimated that approximately 10-12% of the agricultural area is affected by waterlogging or severe soil drainage constraints (Kaur et al., 2020b). In the United States, for example, flooding poses a significant hazard and has been ranked as the second most impactful abiotic stress factor, following drought, in terms of crop production losses over a 12-year period (Kaur et al., 2020b). These constraints pose significant challenges to agricultural productivity and sustainability in affected regions. A long-term research study conducted in China also revealed that the grain yield of wheat cultivars showed a steady improvement over the years. However, it was observed that the rate of yield improvement was lower under waterlogging conditions compared to normal watering conditions. Under normal watering conditions, the grain yield increased by 53 kg ha^−1^ per year (equivalent to a yearly improvement of 0.6%), while under waterlogging treatment, the increase was 35 kg ha^−1^ per year (equivalent to a yearly improvement of 0.51%) from 1967 to 2010 (Ding et al., 2020). The research conducted in China, although specific to the wheat cultivars in that region, provides a general indication that as wheat cultivars are continuously developed and improved over time, there is a tendency for their overall waterlogging tolerance to decline. This observation has broader implications for wheat breeding programs and the selection of traits prioritized during cultivar development.

**Figure 4:**
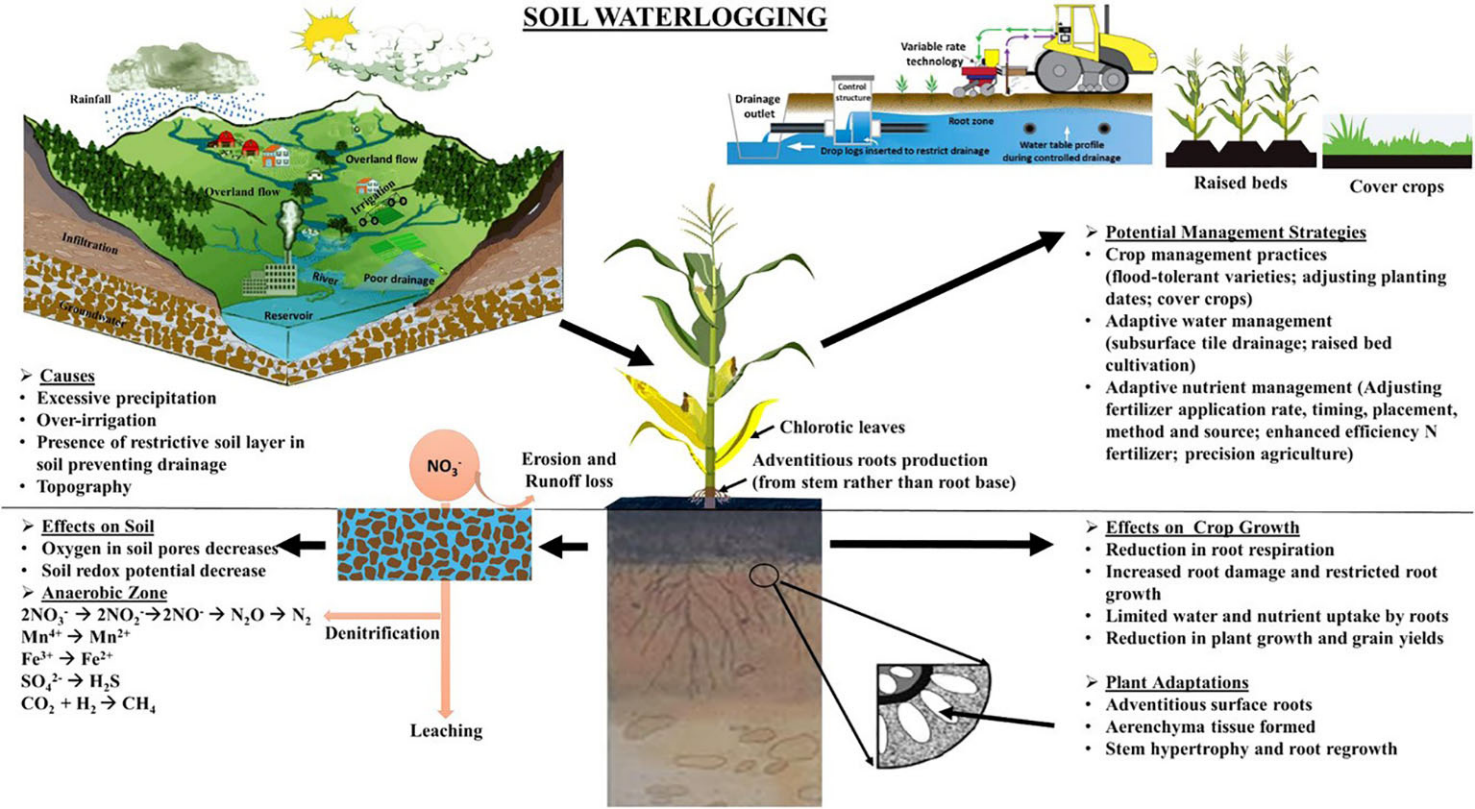
Illustrates a depiction of the causes of soil waterlogging, its impact on crop production nitrogen losses, and potential approaches for managing these issue (Kaur et al., 2020b).

The detrimental impact of waterlogging on the quality, yield, and physiological aspects of wheat universally highlights the need to devise effective mitigation strategies. Addressing these constraints requires a combination of management strategies tailored to a particular or specific conditions. These may include implementing improved drainage systems, raised bed planting, ensuring land leveling, optimizing the sowing period, adaptive nutrient management and utilizing plant growth-promoting substances (**Figure 4**) (Pramanick et al., 2023). The use of raised beds has proven to be significant in high rainfall regions, as observed in Victoria, Australia. The substantial yield increases observed for both wheat (50%) and barley (30%) in Victoria demonstrate the effectiveness of raised beds as an agronomic measure in mitigating the adverse effects of waterlogging in high rainfall areas (Manik et al., 2019). In addition to the agronomic based mitigation interventions, the contemporary advancements in biotechnology, including functional genomics, offer promising approaches to identify specific genes or QTL (Quantitative Trait Loci) associated with waterlogging tolerance in wheat. Genome modification techniques are also employed to enhance wheat’s capacity to withstand waterlogging conditions substances (Pramanick et al., 2023). These biotechnological interventions play a significant role in the development of novel wheat cultivars with improved tolerance to waterlogging.

### 3.5 Biofortification of crops as influenced by the changing climate

The nutritional functional diversity of the cropping system, which is based on both on-farm durum wheat species diversity and nutritional composition, holds significant potential to address malnutrition and associated health complications. However, increasing demand for nutritious, safe, and healthy food because of a growing population and changing climatic conditions; pledge to maintain biodiversity and other resources pose a major challenge to the crop production sector (Dwivedi et al., 2017). Micronutrient deficiency, such as zinc malnutrition, has been observed to affect more than 17% of the world population, and enrichment of the atmospheric carbon dioxide significantly lowers grain zinc concentration by about 9.1% (Soares et al., 2019). This reduction may further affect about 138 million people and place them at a new risk of zinc deficiency by 2050 (Myers et al., 2015; Soares et al., 2019).

**Figure 5:**
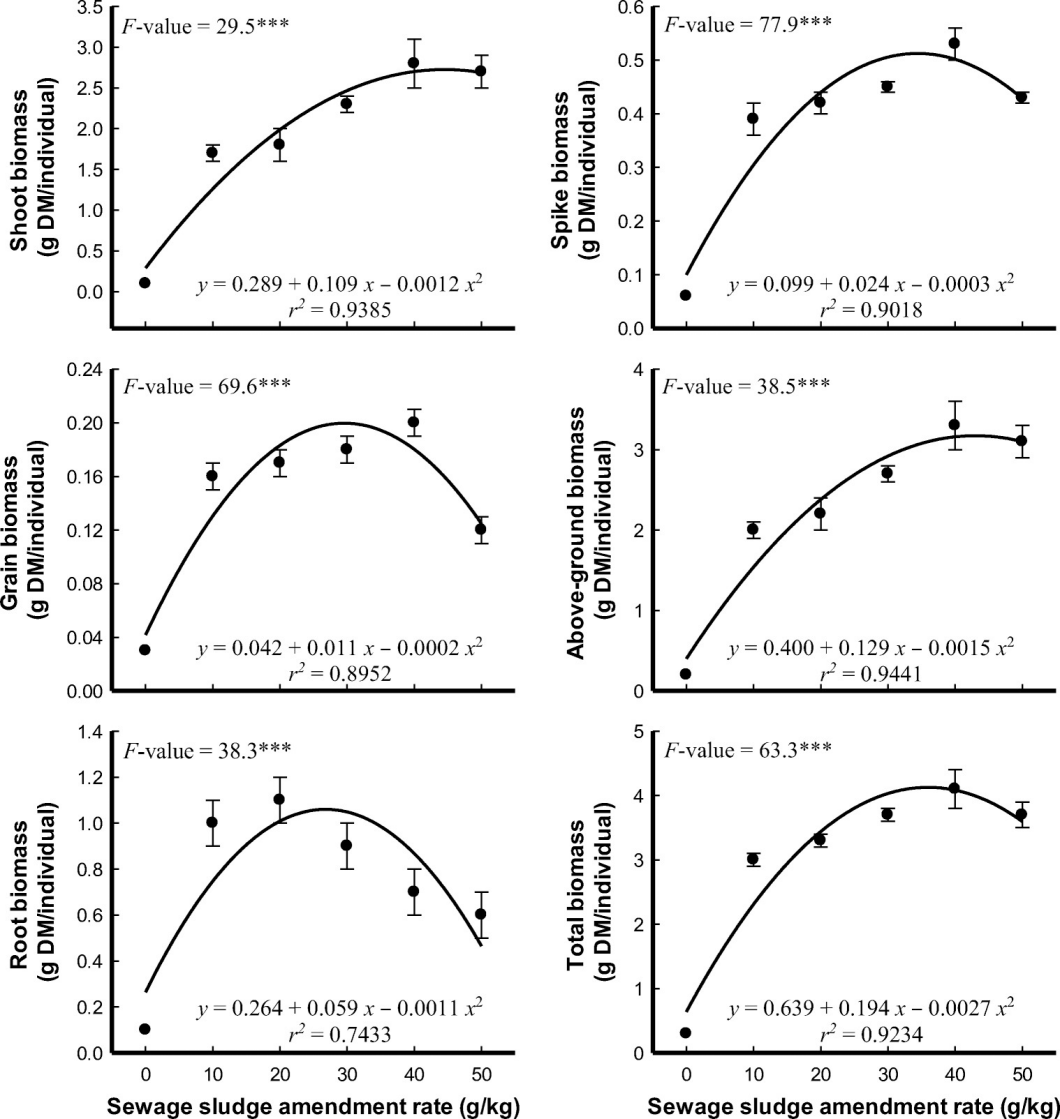
Effects of different sewage sludge amendment rates on the biomass of wheat harvested after 80 days (means ± standard error, n = 24). F values represent a one-way ANOVA and degrees of freedom (df) = 5. ***P<0.001 (Eid et al., 2019).

When wheat crops are grown under elevated CO_2_ conditions, wheat tend to accumulate a higher level of carbohydrate and enhance yield, but with reduced concentrations of certain minerals such as zinc and iron by about 3% and 5%, respectively (Richard et al., 2022). This result highlights that climate change adaptation strategies that benefit grain yield may not always have a positive effect on grain qualitative traits, thus putting further pressure on global quality wheat production (Asseng et al., 2019). A recent meta-analysis also showed a significant decline in grain Zn, Fe, S, Ca, Mg, P, Mn, K, and Mo with increasing CO_2_ concentration (Ben et al., 2021). The decreased grain mineral concentration could be primarily attributed to changes in plant physiology, such as a decrease in the pace of transpiration rate, i.e., linked to stomatal closure due to long-term exposure to elevated CO_2_, since higher CO_2_ could reduce the mass flow in the soil toward roots, which diminishes the availability of mobile minerals in the rhizosphere (Loladze, 2002). However, higher grain zinc and iron deposition has been observed under high temperature conditions, offsetting the decrease in grain mineral concentration due to elevated carbon dioxide (Wang et al., 2020). Although the specific mechanism behind this response is not yet fully understood, it could be attributed to improved drought induced transportation of trace elements, leading to higher grain mineral concentrations (Ge et al., 2010). Addressing these issues generally requires a multifaceted approach, such as breeding crop varieties that are more efficient in micronutrient uptake and allocation, optimizing nutrient management approaches, and considering agronomic practices that can enhance the availability of mineral nutrients in the root zone. It has been also observed that the coexistence of abiotic stress on nutrient composition had a positive effect on gluten, Fe, Zn, and protein, showing respective increases of 19.11%, 14.42%, 7.20%, and 4.60%. However, the decrease in yield offset the concentration gains in other nutrients, leading to a decrease in K (-32.08%), Mn (-21.65%), P (-13.12%), and Mg (-7.66%) (**Figure 5**). This result highlights the trade-off between nutrient yield and overall grain yield when subjected to abiotic stress. While certain nutrients such as gluten, Fe, Zn, and protein showed improvements in yield, the decrease in yield can offset the gains in concentration for other essential nutrients.

## 4 The climate-nutrient nexus: managing nutrient inputs for sustainable agriculture in a changing climate

### 4.1 Nitrogen application under drought condition: from risk to resilience

Nitrogen application under drought conditions is a crucial aspect of agronomic practices, transforming the concept of risk into resilience. Improving crop resistance to heat and drought stressors through plant breeding and adjustments in agronomic practices such as site-specific nutrient management, time of sowing, and proper nitrogen fertilization are thought to be useful in climate change adaptation (Van Ittersum et al., 2013). Nitrogen metabolism such as, ion absorption, nitrogen assimilation, amino acid synthesis, and protein synthesis are very important drought tolerance indicators (Li et al., 2020). Increasing nitrogen application up to a certain level may alleviate drought-induced stress by increasing root osmotic regulators, stimulating the acceleration of root biomass accumulation, and enhancing nitrogen assimilation ability, which would reduce moisture limitations (Xiong et al., 2018). In the presence of mild drought stress, application of nitrogen fertilizer at a higher dose has been demonstrated to enhance the plasticity expression of root development (Tran and Kano-Nakata, 2014). Integrated nitrogen, phosphorus, and potassium applications also profoundly increase osmoprotectant accumulation and activity of both nitrogen absorption and antioxidant enzymes to increase wheat grain production and drought tolerance (Shabbir et al., 2016). However, it is important to note that excessive nitrogen application can have negative impacts on plant growth and development, including reduced drought tolerance (Liu et al., 2016). This suggests that the regulatory function of nitrogen in drought-induced stress tolerance of plants is dependent on the severity of the stress, nitrogen amount, and crop species variation (Xiong et al., 2018). Hence, it is essential to apply nitrogen in appropriate amounts and at the right time to maximize its benefits for the growth and development of durum wheat.

Nitrogen application at the appropriate timing and amount can aid in the development of stress defense mechanisms while also promoting normal crop growth (Chang and Liu, 2016). Under proper nitrogen fertilization, plants can produce adequate antioxidant enzyme activity and osmotic adjustment by generating proline accumulation to alleviate drought-induced physiological damage (Li et al., 2020). In a universal agreement, higher nitrogen metabolism enhances the drought resistance level of crop plants (Zhong and Cao, 2017). It means that higher levels of nitrate transport and assimilation could subsidise to improve the drought induced stress tolerance level of crops (Zhong and Chen, 2015). Hence, implementing innovative agronomic techniques and harnessing the potential of nitrogen-based fertilizers could enhance durum wheat productivity, adapt to challenging climatic conditions, and ultimately transition the crop sector from a vulnerable state to one of resilience. However, to the best of our knowledge, the nitrogen effect under varying drought stress levels at various intensities of abiotic stress and the phenological plasticity of durum wheat are not clearly understood, which may require a comprehensive investigation at a refined molecular level.

### 4.2 Harnessing nanoparticles: revolutionary solutions for mitigating crop abiotic stress

The past few decades have seen significant structural modifications in agricultural cultivation systems that are intended to improve how crops respond to diverse abiotic stresses. The use of nanoparticles in agricultural production systems has been observed as advantageous for both environmental stewardship and crop productivity enhancement. Nanoparticles are small molecular aggregates with dimensions of 1–100 nm. These tiny particles may easily enter the plant cells through both above-ground organs, such as the cuticle, epidermis, stomata, and hydathodes; and underground organs, including root tips, cortex, lateral roots, and wounds (El-Saadony et al., 2022). Due to their high reactivity, nanomaterials exhibit efficient nutrient absorption for plants, resulting in greater utilization efficacy and minimal losses in comparison to conventional fertilizers (Avila-Quezada et al., 2022).

Nanoparticles have been frequently reported to improve growth, development, grain quality, and yield of crops under a range of abiotic stress conditions. It has been observed that a proper soil-based application of analcite nanoparticles enhances the germination and morphometric traits of wheat, particularly under dry climatic conditions (Hossain et al., 2021). Improved germination percentages have also been observed in other crops following the application of ZnO-based nanoparticles (Sedghi et al., 2013). Nanoparticles such as Cu-based fertilization influence various physio-morphological traits such as biomass yield, chlorophyll concentration, carotenoid contents, leaf water content, and anthocyanin, particularly under dry climatic conditions (van Nguyen et al., 2022). An increase in wheat morphometric traits due to the application of Cu and Zn nanoparticles could be due to improvements in antioxidant enzyme activity and relative moisture content, which ultimately reduce the effects of drought stress (Taran et al., 2017; Semida et al., 2021). Through adjusting these processes, nanoparticle-based fertilization could help crops adapt to drought stress and maintain both yield and grain protein concentration in the current climate change scenarios.

Under drought conditions, the application of silicon dioxide (SiO_2_) nanoparticles has been found to increase the shoot length, and relative water content while reducing superoxide radical formation, and membrane damage (Turgeon, 2010). This is attributed to the ability of SiO_2_ nanoparticles to improve water uptake and retention, thus reducing the negative impacts of water stress on the growth, development, and grain quality of crops. When silicon dioxide (SiO_2_) and titanium dioxide (TiO_2_) nanoparticles are simultaneously applied, significant improvements in grain yield and stress tolerance levels of crops have been observed (Shallan et al., 2016). The enhanced grain yield following SiO_2_ fertilization can be attributed to various factors, including improved photosynthesis, stomatal conductance, and stress tolerance of crops (Ashkavand et al., 2015). However, the effectiveness of nanoparticle fertilization in enhancing drought tolerance and improving grain yield can be influenced by nutrient application methods. It has been observed in some studies that foliar application of titanium dioxide nanoparticles can enhance grain yield and stress tolerance in wheat more effectively than other application methods (Jaberzadeh et al., 2013). Additionally, the application of zinc-based nanoparticles has shown potential for increasing grain zinc concentration along with improving grain yield, proline, glycine betaine, free amino acids, protein content, and other yield-related traits (Dapkekar et al., 2018; Ghani et al., 2022). Zinc-based nanoparticle applications have additional effects beyond the nutrient supply, such as enhanced nutrient uptake, increased photosynthetic activity, and improved water use efficiency; these specific effects can contribute to the overall improvement of grain yield, protein content, carbohydrate metabolism, and other yield-related traits (Verma et al., 2022a).

Drought stress could also negatively affect the nutrient absorption and utilization efficiency of plants, including essential nutrients like nitrogen, phosphorus, and potassium (Raza et al., 2023). However, the application of silicon-based nanofertilizers has been shown to have positive effects on soil nutrient availability, including nitrogen, phosphorus, and potassium (Rizwan et al., 2019). This effect ensures that crops have access to necessary essential nutrients, particularly in water-limited environments, stimulating plant growth, yield, and grain protein content. It has also been shown to have beneficial effects in reducing the accumulation of heavy metals, such as cadmium (Cd), under drought conditions and improving the drought tolerance of crops by initiating different pathways (Khan et al., 2019). It is also worth mentioning that climatic extremes such as drought, salt, and waterlogging could enhance the production of reactive oxygen species (ROS), leading to oxidative stress (Hasanuzzaman et al., 2020). This action impaired water uptake, disrupting biological membranes, ionic imbalance, oxidative damage and nutritional imbalance, reducing cell division and expansion, lipid metabolism rate of photosynthesis and consequently impairing yield attribute traits (Kumari et al., 2022). Through neutralizing ROS and stabilizing cell membranes, silicon-based nanoparticles could contribute to reducing the damaging effects of ROS on wheat cells (Hussain et al., 2019). This could have a significant positive effect on durum wheat physiology, such as enhanced stress tolerance, improved crop health, and vigorous growth.

The other important nutrient that has gained attention as a mitigation solution for climate change is nitrogen-based nanoparticles due to their ability to enhance nitrogen use efficiency and reduce nitrogen losses. It has been shown to offer potential positive benefits in terms of tillering capacity, crop health, vigorous growth, and leaf colour changes, i.e., from light yellow to green (Kumar et al., 2022a). Alteration of the life colour impels that adequate application of nitrogen based nanoparticles could maintain the greenness of crops, which caused the crop to mature at its proper time and promoted proper development of the grains, and grain protein content remained high. Nitrogen-based fertilization also improves antioxidant defense mechanisms and reduces drought-induced oxidative damage by promoting the synthesis and activity of antioxidant enzymes such as superoxide dismutase (SOD) and peroxidase (POD), thus improving photosynthesis and crop stress tolerance levels (Raza et al., 2022). The improved photosynthesis could, in turn, increase carbon assimilation and energy availability, which are important for crop growth and drought stress tolerance. Although nanoparticles have a positive effect on overall crop productivity, the optimum concentration and sources of nanoparticles may vary depending on specific growing conditions and wheat varieties. Thus, extensive and rigorous field trials are necessary before widespread adaptation of nanoparticle-based fertilization approaches aimed at improving crop yield and grain protein content under changing climate scenarios.

### 4.3 Revitalizing agriculture with silicon-based fertilizers for enhanced productivity and environmental sustainability

Revitalizing agriculture through the use of silicon-based fertilizers has emerged as a promising strategy to support overall growth and development of crops, such as by enhancing photosynthetic efficiency and limiting electrolytic leakage under changing climatic conditions (Mir et al., 2022). Although it is not classified as an essential nutrient for all crops, a number of reports indicate the critical role of silicon in enhancing crop abiotic and biotic stress tolerance, such as drought, salt, freezing, nutrient imbalance, and radiation damage (Wang M. et al., 2021). Application of silicon under drought conditions has increased the photosynthesis rate, stomatal conductance, and antioxidant defense compared to plants grown without silicon application, which leads to efficient energy conversion and increased biomass production (Ali et al., 2019). It also enhances the crop’s ability to withstand drought stress by maintaining root growth and improving water transport (Yan et al., 2016). The effects of which are attributed to the increased antioxidant defense and decreased oxidative stress induced by silicon fertilization (Gunes et al., 2007). This means that proper application of silicon-based fertilizers could allow wheat crops to explore or access water from deeper soil layers in a water-limited environment.

Through enhancing photosynthetic activity, increasing the efficiency of nutrient uptake, delaying senescence, improving stomatal responses, and enhancing drought tolerance silicon fertilization enables plants to maintain higher biomass and grain yield production under abiotic stress condition (Schaller et al., 2021; Irfan et al., 2023). Improved morphometric and physiological traits, such as gas exchange capacity, total root length, surface area of the root, volume of the root, and plant height, dry matter, have been observed following silicon application under drought conditions (Irfan et al., 2023; Chen et al., 2011). A larger silicon mediated root surface area could facilitate enhanced water and nutrient absorption, which is particularly important when soil moisture availability is limited. This silicon-mediated change in root development could further improve root endodermal silicification and suberization (Fleck et al., 2011). The enhanced silicification of the endodermis could contribute to improved water and nutrient uptake efficiency by restricting the passive flow of water and solutes.

While the exact mechanisms of how silicon enhances seed germination under drought conditions are still being studied, several research studies have reported positive effects on the stimulation of seed germination and spikelet sterility in wheat (Hameed et al., 2013). In addition to its effect on grain yield, proper fertilization with silicon could maintain or even increase the protein content of durum wheat under drought conditions (Kutasy et al., 2023). Because, silicon enhances the activity of enzymes involved in nitrogen metabolism, this leads to improved nitrogen uptake, assimilation, and translocation within the plant. This, in turn, can contribute to higher grain protein accumulation. Hence, the positive effects of silicon on physiology, yield formation, nutrient uptake, and grain quality substantiate the need to include these essential nutrients in the cultivation system of durum wheat under changing climatic conditions. An intimation to adapt silicon as a remedial measure under changing climatic conditions, is evident from the upregulation of genes involved in adaptation mechanisms such as phytohormone metabolism and cell wall synthesis upon supplementation of silicon fertilizers (Haddad et al., 2019).

The presence of silicon in plant tissues can enhance the crop’s ability to withstand drought stress through several mechanisms, such as the antioxidant system and reducing drought-induced oxidative stress (Gunes et al., 2007). It has been observed that the application of silicon-based fertilizers exhibits improved wheat resistance to drought stress, particularly in silicon-accumulating varieties compared to non-silicon-accumulating crops (Thorne et al., 2021). However, in plant species that are less effective at accumulating silicon, the application of silicon can still have positive effects in countering drought stress, such as tomato and canola (Wang M. et al., 2021). These suggest that both accumulators and non-accumulators could benefit from silicon fertilization, and even low levels of Si accumulation can contribute to improved plant performance under drought conditions (Katz, 2014). A higher drought tolerance level in silicon-accumulated varieties could be due to reduced transpiration, increased water uptake, regulating stomatal behaviour, enhanced antioxidant activity, and improved photosynthesis following silicon application (Wang M. et al., 2021). ). These traits could therefore contribute to the ability of silicon-accumulated wheat varieties to better withstand and recover from abiotic stress, ultimately leading to improved grain yield and resilience in water-limited environments.

### 4.4 From waste management to climate solutions: sewage sludge fertilization as an adaptive agricultural approach

The application of sewage sludge could be considered an adaptive agricultural approach with potential benefits for both waste management and climate solutions. In the presence of abiotic stress, such as drought stress, the availability of water is very limited, and crops like durum wheat may not be able to absorb nutrients from the soil effectively. The use of sewage sludge has been found to improve the water-holding capacity of soil, which can improve the seasonal drought stress tolerance of durum wheat (Boudjabi et al., 2015; Debiase et al., 2016). In recent years, sewage sludge fertilization has gained interest and recognition due to its nutrient content, organic matter contribution, and cost-effectiveness in comparison to synthetic fertilizers (Inmaculada et al., 2007; Boudjabi et al., 2015). It contains substantial amounts of nutrients essential for plant growth, including nitrogen, phosphorus, and organic carbon (Fytili and Zabaniotou, 2008). Through improving nutrient availability and mitigating the effects of drought stress, sewage sludge fertilization can also contribute to an increase in grain protein, and amino acids content, resulting in improved nutritional quality of wheat (Mazen et al., 2010).

**Figure 6:**
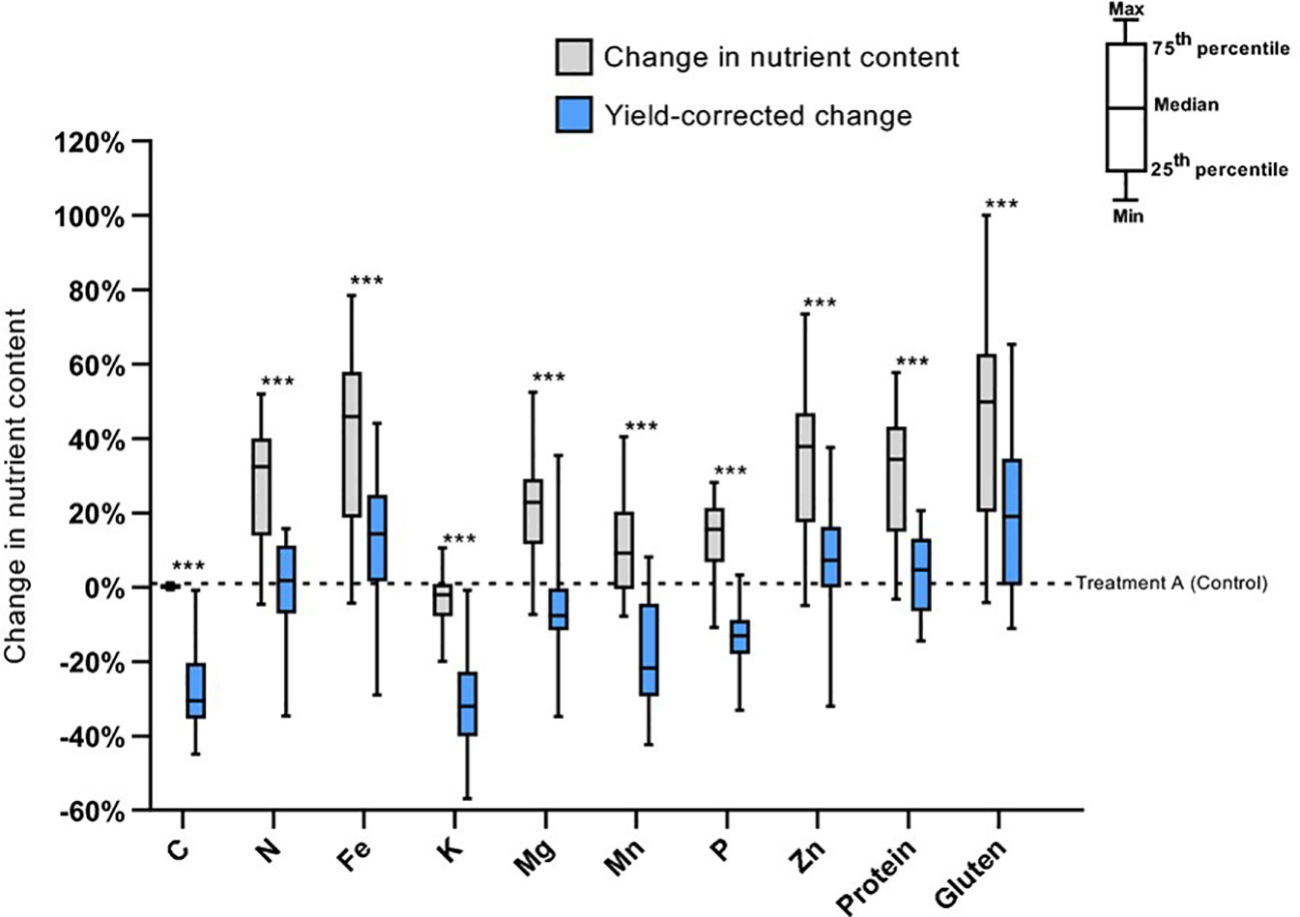
Illustrates the average percentage change in nutritional composition and yield associated nutrient content of wheat varieties under the co-existence of abiotic stress compared to the control. NB: “A” stands for ambient CO2, lower temperature settings, and no O3 addition (control). The bars indicated with *** refer to a significant difference at p = 0.001 between the normal and yield-corrected nutrient content (Galani et al., 2022).

When applied as fertilizer, sewage sludge releases nutrients gradually, ensuring sustained supply to the crops over an extended growing season (Muter et al., 2022). The slow release of sewage sludge fertilizers could be more advantageous, particularly in dry land farming where drought is a serious factor, as it allows plants to access essential nutrients steadily in a water-limited environment (Muter et al., 2022). Additionally, augmenting sewage sludge fertilizers has improved the grain yield of durum wheat, through which sludge-based fertilization allows root growth and helps to explore deep into the soil and absorb more water, thus enabling the crop to avoid the seasonal water stress effect (Sonia et al., 2019). Improved water utilization efficiency could help durum wheat plants maintain physiological processes, minimize stress, and allocate resources efficiently. The water stress reduction role of sewage sludge application has been confirmed, through which sludge based fertilization improves water retention capacity, regulates chlorophyll a, and enhances aboveground biomass production (Boudjabi et al., 2015). However, when sewage sludge is applied in excess, it could increase osmotic stress, perhaps due to the hydrophilic effect of organic matter contained in the sludge (Boudjabi et al., 2015). Higher growth parameters and biomass yield have been observed (**Figure 6**), particularly under low concentrations of sludge application, which further decreases the risk of heavy metal toxicity (Eid et al., 2019). Inversely, the application of sewage sludge-based fertilization, particularly at higher doses, have showed a synergistic effect that mitigates drought stress in other crop plants. This effect is attributed to the increased accumulation of osmoregulators, which assist plants in coping with water scarcity (Oustani et al., 2015). However, responsible application practices should be followed to ensure the safe and effective utilization of sewage sludge as a fertilizer (Oustani et al., 2015). These results universally indicated that improper or excessive application of sewage sludge can have a negative consequence including nutrient imbalance, heavy metal accumulation, and environmental pollution. Hence, regular monitoring soil and plant samples can provide valuable insights into nutrient levels and potential heavy metal accumulations of wheat, enabling timely corrective measures if needed (Feszterová et al., 2021).

The use of sewage sludge fertilizers can have significant agronomic benefits, such as providing nutrients such as nitrogen, ammonium, potassium, and zinc and thus improving soil quality (Marin and Rusănescu, 2023). When sewage sludge is applied to the soil, it can indeed improve soil organic matter (SOM) content, nutrient contents, soil porosity, bulk density, aggregate stability, and available water holding capacity (Simões-Mota et al., 2022). This improvement could be attributed to several factors, such as the high moisture content of sewage sludge and the presence of soil organic matter (Achkir et al., 2023). This can be particularly beneficial in drought conditions, as the soil can hold more moisture, providing a reservoir for plant growth and reducing seasonal water stress. However, the presence of higher organic matter in sewage sludge can stimulate microbial populations, leading to increased microbial respiration and subsequent water consumption (Hechmi et al., 2020). It clearly indicates that if the soil is already experiencing drought conditions, this increased demand for moisture could further stress crops and reduce their growth and yield potential. Hence, it is very important to consider the balance between the positive effects of sewage sludge application on moisture retention and the potential negative consequences associated with increased microbial activity and soil moisture demand, particularly under drought conditions.

The application of sewage sludge can also provide cost benefits by reducing the need for mineral fertilizers and promoting economically beneficial crop production (Silva et al., 2022). Previous studies have shown that the application of sewage sludge can save up to 25% of mineral fertilizer requirements, and its combined application with inorganic fertilizers proved economically beneficial for cereal production (Ankush et al., 2021). While sewage sludge can provide benefits as a fertilizer, it is important to consider and address the potential risks associated with its use. Because sewage sludge may contain heavy metals, and organic pollutants that accumulate in soils and transfer to crops and groundwater if not properly managed (Healy et al., 2016).

### 4.5 Zinc-infused fertilizers: cultivating resilient harvests amidst changing climate

The need for improved compatibility between food security and environmental stewardship is of considerable urgency under the current climate change scenarios. Several studies have shown that zinc deficiency can result in reduced crop yields and lower protein content in grain (Melash et al., 2019). This is because zinc is necessary for the synthesis of enzymes that are involved in nitrogen metabolism, which is essential for protein production in crops. Under changing climate conditions, such as increased temperature and rainfall variability, crop growth and development can be negatively affected. However, the use of zinc-containing fertilizers has been shown to mitigate some of the negative effects of changing climate conditions on crop yields and grain protein content (Tao et al., 2018). These effect could be achieved through supporting nutrient limitations and physiological process such as improved photosynthesis pigment, active oxygen scavenging substances, and a reduction in lipid peroxidation under drought condition (Ma et al., 2017). This implies a huge grain yield and quality reduction would occur under the co-existence of soil zinc deficiency, and drought conditions have been observed to have a more profound effect on decreasing the yield and quality of wheat grain (Bagci et al., 2007).

Drought stress could be alleviated through the proper application of zinc-containing fertilizers, and this effect may be attributed to its ability to detoxify reactive oxygen species (ROS) generation and increase antioxidant enzymes (Wang and Jin, 2007; Sofy, 2015). It has been also observed that the application of various levels of zinc resulted in a significant increase in catalase, superoxide dismutase, peroxidase, and ascorbate peroxidase activities at 40% water holding capacity, where water availability is limited (Sattar et al., 2022). This result suggests that zinc fertilization can enhance the antioxidant defense mechanism of wheat crops, enabling them to better cope with water deficit-induced oxidative stress. However, the effectiveness of zinc-containing fertilization can vary depending on the method of application, particularly in enhancing the drought tolerance level and yield performance of wheat crops. The use of zinc priming alone or in amalgamation with zinc foliar-based application has been found to improve the regulated dissipation of excess energy in wheat. This effect was profound, with zinc priming alone increasing the regulated dissipation by over two-fold, and when coupled with zinc foliar application, the increase reaches three-fold under drought conditions (Pavia et al., 2019). Late-season foliar application of zinc under drought conditions could also improve photosynthesis activity, pollen viability, the number of fertile spikes, the number of grains per spike, the water-use efficiency of the wheat crop, and grain zinc concentration (Karim et al., 2012). Applying zinc-based fertilizer has also improved yield and grain zinc concentration by about 10.5 and 15.8%, 22.6 and 9.7%, and 28.2 and 32.8% under adequate water supply, moderate drought, and severe drought, respectively (Ma et al., 2017). The enhancement in yield and grain zinc concentration was maximum under severe drought conditions, which implies that soil-based zinc fertilization may be particularly beneficial in improving wheat crop performance during periods of drought stress.

### 4.6 Seaweed extracts and climate resilience: how marine-based fertilizers can strengthen agricultural systems

The modern crop production system is facing a critical challenge due to extreme environmental changes. These changes are posing a serious challenge to the crop production sector, necessitating the development of trailblazing strategies for sustainable agriculture and food security. The use of seaweed extracts as a natural and sustainable plant growth promoter is gaining increasing attention, in recent years. It contains various compounds such as cytokinins, auxins, and betaines that have been shown to improve the water-use efficiency and stress tolerance of crops (Ali et al., 2022). The seaweed extracts are also abundant in phytohormones, sterols, carbohydrates, polysaccharides, sugars, polyphenols, vitamins, lipids, amino acids, peptides, proteins, macronutrients, and micronutrients that can potentially enhance plant growth and yield (Shukla et al., 2019). These substances could improve photosynthesis activities, nutrient uptake, resiliency, crop development, and soil health, allowing crops to better withstand drought conditions (Deolu-Ajayi et al., 2022). Seaweed extracts have been found to enhance root growth, carotenoids, and tissue water content, which can help crops access water and essential nutrients more efficiently under dry climatic conditions (Ali et al., 2022). An increase in the aggregation of soil particles, soil nutrient availability, aeration, and water-holding capacity has been observed following the application of soluble alginates from seaweeds and protein hydrolysates (Colla et al., 2017).

The application of seaweed extracts improves the drought tolerance of wheat by improving water retention capacity, enhancing root growth, and increasing photosynthesis activity (Sharma et al., 2019). The ability of seaweed extracts to enhance the antioxidant activity of reactive oxygen species scavenging enzymes, such as superoxide dismutase, peroxidases, catalases, and phenolic antioxidants could explain the potential of seaweed application to improve crop stress tolerance (Kumar et al., 2013; Deolu-Ajayi et al., 2022). Important physiological traits such as chlorophyll content were also improved following the application of seaweed extracts by ameliorating the biogenesis of chloroplasts and decreasing chlorophyll degradation (Jannin et al., 2013). This effect could be due to the up-regulated genes that are strongly linked with photosynthesis, cell metabolism, stress response, and S and N metabolism (Jannin et al., 2013). This result universally implies that by protecting photosynthetic tissues from damage, seaweed extracts can help durum wheat varieties maintain and produce energy from sunlight, even in water-scarce environments. Although, effectiveness of seaweed extracts and their method of action in crops are still not well understood, the application of seaweed extracts in stimulating yield, promoting vegetative growth, and ameliorating grain protein content under stress conditions indicates that it is more essential to encourage adoption by durum wheat producing farmers, particularly in drought-prone areas.

The seaweed extract can maintain the water balance of crops and reduce water loss through transpiration. Studies have shown that application of seaweed extracts under drought conditions can improve wheat grain yield by up to 8.04% (Najafi Vafa et al., 2022). This could be due to enhanced crop drought tolerance through improving water retention capacity, reducing transpiration, and enhancing the activity of antioxidant enzymes that protect crops against drought-induced oxidative stress. A vital function of seaweed extracts in maintaining absorption of soil nutrients by crops, stimulated the growth and enhanced plant resistance to abiotic stress could be the cause of such a huge yield advantage (Chen et al., 2021). It has also been observed that the application of seaweed extracts increases the freezing tolerance of crops other than wheat, such as barley, with an increase in winter hardiness (Ganesan et al., 2015). The attenuation effect of seaweed extracts against drought, cold, and salinity stress effects has been shown to be mediated through enhanced root morphology, a build-up of non-structural carbohydrates, which improved storage of energy, enhanced metabolism, and water adjustments, as well as the build-up of proline (Ganesan et al., 2015).

When wheat is grown under elevated atmospheric carbon dioxide, the crop tends to allocate more resources to photosynthesis and less to nitrogen composition, which can result in decreased grain protein content (Tcherkez et al., 2020). Offsetting resource allocation between photosynthesis and protein synthesis has been observed following the application of seaweed extracts under certain conditions, resulting in higher storage proteins in the grain (Deolu-Ajayi et al., 2022). Additionally, the application of seaweed extracts can also improve grain yield and protein content in crops grown under waterlogged conditions. This is thought to be due to stimulating water retention, soil aeration, and nutrient availability, thereby promoting grain nutritional composition (Deolu-Ajayi et al., 2022). Priming wheat seeds with extracts of U. linza or C. officinalis has shown positive effects on chlorophyll content, carotenoid levels, sugar accumulation, protein synthesis, and lipid metabolism (Arun et al., 2023). Seaweed priming enhances protein and sugar contents by facilitating the absorption of major elements, notably magnesium, which activates chlorophyll synthesis and boosts photosynthetic rates (Hamouda et al., 2022).

The foliar spray of seaweed extract has been reported to be effective in improving the performance of wheat varieties under drought conditions. This treatment has been correlated with numerous positive effects, such as the improvement of compatible osmolytes, antioxidant compounds, and genetic variation in non-coding chloroplast DNA regions like the trnL intron and psbA-tnH (Ali et al., 2022). This suggests that seaweed extracts could be a promising agronomic strategy for improving the drought tolerance of crops, which is becoming increasingly important under the current climate change scenarios and water scarcity. A number of studies further suggest that seaweed extracts may increase the production of stress response genes in plants through several mechanisms, such as hormonal regulation, antioxidant activity, and enhanced nutrient availability. Enhanced stress response genes such as Na+/K+ transporters and late embryogenesis abundant (LEA) proteins, including dehydrins, and aquaporins, have been observed following seaweed extract application under abiotic stress conditions (Goñi et al., 2018; Rasul et al., 2021). Although the precise mechanism by which seaweed extracts increase the production of stress response genes in plants is not yet fully understood and may depend on the specific extract and varieties used, these mechanisms suggest that seaweed extracts can provide multiple benefits to crops under stress conditions.

## 5 The feasibility, environmental risk, and mitigation strategies of nutrient-based climate interventions

Nutrient based climate change interventions in agriculture have gained attention as potential strategies to mitigate greenhouse gas emissions and adapt to changing climatic conditions (Kumar et al., 2022b). These interventions involve application of fertilizers, and altering agricultural practices to enhance carbon sequestration, nutrient availability, reduce nitrous oxide, and improve overall soil health (Hassan et al., 2022b). However, prior to implementing such interventions on large scale, it is crucial to thoroughly asses their feasibility, potential environmental risk, and develop effective mitigation strategies to ensure long term sustainability. When considering the feasibility and environmental risks of using nutrients such as silicon, sewage sludge, zinc and sulphur for climate interventions, it is also essential to evaluate factors such as nutrient availability, local regulations and guidelines, potential impacts on water quality and soil health, and overall sustainability of the practices. Implementing these interventions should be done with careful planning, monitoring, and adherence to best management practices to minimize any negative environmental consequences (Muter et al., 2022).

The environmental implications of sewage sludge (SS) in various disposal scenarios, such as landfill disposal, agricultural use, and other applications, have garnered significant attention. The energy consumption during the treatment of SS is the primary contributor to global warming, accounting for over 50% of the impact (Muter et al., 2022). The disposal of sludge in agricultural areas primarily contributes to terrestrial acidification, and freshwater ecotoxicity, global warming, eutrophication, and acidification (Rorat et al., 2019). Additionally, the transportation of SS to agricultural areas has been identified as a significant factor influencing terrestrial and freshwater ecotoxicity, as well as ozone formation in terrestrial ecosystems. The toxicity associated with SS is often linked to the presence of toxic heavy metals such as Cr, Pb, Ni, Hg and Cd because industrial wastewater is mixed with sewage (Mañas and de las Heras, 2021; Muter et al., 2022). Hence, the selection of an appropriate waste treatment method plays a crucial role in mitigating the environmental impact associated with sewage sludge application. Various methods, including anaerobic digestion, pyrolysis, and supercritical water oxidation, have been identified as effective approaches for reducing the environmental risks associated with SS (Teoh and Li, 2020). Anaerobic digestion, for instance, allows for the conversion of organic matter in SS into biogas, cost effective, minimizing greenhouse gas emissions and reducing the potential for global warming (Rorat et al., 2019; Teoh and Li, 2020). Additionally, composting or co-composting with other biodegradable wastes and additives is an important treatment method for SS, enabling a significant reduction in volume and minimal emissions of hazardous substances, making it environmentally acceptable when compared to incineration (Sugurbekova et al., 2023). It has been also observed that applying sewage sludge at lower doses presents minimal risks to the environment, while simultaneously enhancing the grain yield and quality of crops. Hence, carefully determining the appropriate dosage, the potential negative impacts associated with SS application can be mitigated, ensuring that the benefits outweigh the risks.

Although further research is needed to fully understand the long-term effects of nanoparticles, NPs based crop fertilization possess distinct characteristics in comparison to conventional fertilizers (Lina et al., 2023). These unique attributes contribute to a gradual and sustainable absorption of nutrients by crops, primarily because of their high surface-to-volume ratio and reduced nutrient loss (Tarafder et al., 2020). In compression, the conventional fertilizer applications, such as nitrogenous, phosphates, and potassium-based have been found to have low efficiency rates, with nitrogenous fertilizers ranging from 20 to 50%, phosphates ranging from 10 to 25%, and potassium ranging from 35 to 40% (Avila-Quezada et al., 2022). This inefficiency can lead to a significant volume of fertilizers being applied in agricultural practices. However, nanofertilizers offer active sites that facilitate a greater number of biological activities, thereby enhancing the efficiency of nutrient absorption by plants (Feregrino-Pérez et al., 2018). Moreover, NPs also contribute to the improvement of soil fertility and create a favourable environment for the growth of beneficial microorganisms within the soil (Thavaseelan and Priyadarshana, 2021). As a result, nanofertilizers provide sustainable solutions to address issues of environmental pollution and climate change (Solanki et al., 2015). Additionally, the use of NFs presents economic benefits by minimizing the leaching and volatilization of conventional fertilizers. Leaching and volatilization contribute to nutrient loss and environmental pollution, thus reducing these processes could offer a cleaner technology for the environment and provide an attractive proposition for agricultural producers (Avila-Quezada et al., 2022). However, if nanoparticles are not properly managed NFs could adversely affect plants through multiple mechanisms. These include DNA damage, the formation of reactive oxygen species (ROS), interaction with nuclear proteins, chromosomal abnormalities, a decrease in DNA repair mechanisms, and the occurrence of genetic defects. For instance, studies have shown that NiO NPs can penetrate the DNA of plants, causing irreversible damage to their cells (Faisal et al., 2013; Verma et al., 2022b). Similarly, when Co_3_O_4_ NPs were applied to crops, it resulted in apoptosis (cell death) in their cells (Faisal et al., 2016). Furthermore, the use of ZnO NPs has been found to have detrimental effects on the membrane integrity, chromosomal structure, and DNA strand stability in various plant species (Faisal et al., 2016; Bhardwaj et al., 2022). Despite the remarkable efficiency and ease of application, nanofertilizers are accompanied by certain limitations, including complicated production processes, fragile transport, and dosage-sensitive efficiency, which are currently impeding the widespread adoption of nanofertilizers in agriculture (Kalwani et al., 2022). Nevertheless, concerning economic feasibility, nanoparticles have the potential to be economically viable and less environmentally toxic compared to some other alternatives (Upadhayay et al., 2023).

While nutrient based climates change interventions offer potential benefits, nutrients could also carry certain environmental risks that needs to be managed. Application of nutrients, such as silicon, and zinc-based fertilizers are an essential for plants, and silicon-based fertilizers have been developed to enhance plant growth, increase resistance to pests and diseases, and improve abiotic stress tolerance. Silicon inhibits the toxicity caused by heavy metals, protecting plants from their detrimental effects and plays a crucial role in activating soil phosphorus (P), making it more readily available for plants (Imran et al., 2021). This activation process enhances the absorption of P by plant roots, along with other essential nutrients. The positive influence of Si in enhancing crop yield, improving crop resilience, and addressing the challenges of sustainable agriculture and food provision, emphasizing the need for its wider adoption in modern agriculture (Barão, 2023). However, it is important to consider the potential for silicon accumulation in soil, which may affect soil properties and nutrient availability. The application of zinc containing fertilizers in crops has been found to yield several beneficial effects. These advantages include enhanced zinc grain accumulation and protection against cadmium (Cd) uptake and transfer through the roots and xylem-to-phloem pathways (Hassan et al., 2022a). While zinc is an essential micronutrient for plants and offers numerous benefits when applied in appropriate doses, excessive levels can negatively impact foliage and crop yields (Xu et al., 2021). Additionally, improper application can result in zinc leaching into water bodies, causing water pollution (Singh et al., 2022). Hence, proper dosage and application practices, along with regular soil testing, are necessary to prevent the adverse effects associated with zinc-infused fertilizers. In recent years, unmanned aerial vehicle-based spraying (UAV-based spraying) has emerged as a safer, cleaner, and more efficient method for the targeted application of zinc-containing fertilizers. This technology reduces zinc input, increases the recovery rate, and minimizes the risk of fertilizer residue (Xu et al., 2021). This technology expands the options for fertilizing crops and facilitates the production of highly Zn-biofortified grain while optimizing input costs for farmers.

## 6 Conclusions

The changing climate poses a significant threat to crops, including durum wheat, and sustainable adaptation strategies are necessary to maintain food and nutritional security. This comprehensive review article provides a comprehensive overview of the current understanding of nutrient management in durum wheat cultivation under changing climatic conditions. Identifying knowledge gaps and exploring advanced strategies, contribute to the existing literature and provide valuable insights for researchers, agronomists, and farmers alike. It is crucial for future research to focus on investigating the specific nutrient requirements of durum wheat under different climate scenarios and evaluating the effectiveness of innovative nutrient management practices to ensure sustainable and resilient wheat production. Implementing precision farming techniques, optimizing fertilizer application rates and timing, and utilizing precision nutrient delivery systems are potential strategies to maximize farm profitability, efficiency and mitigate the adverse effects of climate variability on durum wheat production. However, the coexistence effect of climatic parameters on nutrient uptake, translocation, grain quality, yield and assimilation mechanisms within durum wheat crops remains poorly understood. Therefore, future research should focus on unraveling these intricacies to develop targeted nutrient management strategies for maximizing grain quality and yield. As the traditional nutrient management practices are also insufficient in addressing the complex challenges posed by climate change, there is a need for advanced nutrient management strategies to mitigate the negative impacts of the changing climate conditions on durum wheat. Hence, adopting innovative approaches such as precision agriculture, controlled-release fertilizers, and site-specific nutrient management can optimize nutrient availability, uptake efficiency, and utilization by durum wheat plants. A continuous research, technological advancements, and farmer education are key to successfully addressing these challenges and realizing the benefits of nutrient management in durum wheat production.

## Author contributions

All authors listed have made a substantial, direct, and intellectual contribution to the work, and approved it for publication. All authors contributed to the article and approved the submitted version.
